# Intramedullary screw fixation with bone autografting to treat proximal fifth metatarsal metaphyseal-diaphyseal fracture in athletes: a case series

**DOI:** 10.1186/1758-2555-4-25

**Published:** 2012-07-20

**Authors:** Sachiyuki Tsukada, Hiroo Ikeda, Yoshie Seki, Masayuki Shimaya, Akiho Hoshino, Sadao Niga

**Affiliations:** 1 Department of Orthopedic Surgery, Kawaguchi Kogyo General Hospital, 1-18-15 Aoki, Kawaguchi, Saitama 332-0031, Japan; 2 Urawa Red Diamonds, 3-4 Ohara, Urawa-ku, Saitama, Saitama 330-0046, Japan

**Keywords:** Jones fracture, Proximal diaphyseal fractures, Bone graft, Thermal necrosis, Torg classification

## Abstract

**Background:**

Delayed unions or refractures are not rare following surgical treatment for proximal fifth metatarsal metaphyseal-diaphyseal fractures. Intramedullary screw fixation with bone autografting has the potential to resolve the issue. The purpose of this study was to evaluate the result of the procedure.

**Methods:**

The authors retrospectively reviewed 15 athletes who underwent surgical treatment for proximal fifth metatarsal metaphyseal-diaphyseal fracture. Surgery involved intramedullary cannulated cancellous screw fixation after curettage of the fracture site, followed by bone autografting. Postoperatively, patients remain non weight-bearing in a splint or cast for two weeks and without immobilization for an additional two weeks. Full weight-bearing was allowed six weeks postoperatively. Running was permitted after radiographic bone union, and return-to-play was approved after gradually increasing the intensity.

**Results:**

All patients returned to their previous level of athletic competition. Mean times to bone union, initiation of running, and return-to-play were 8.4, 8.8, and 12.1 weeks, respectively. Although no delayed unions or refractures was observed, distal diaphyseal stress fractures at the distal tip of the screw occurred in two patients and a thermal necrosis of skin occurred in one patient.

**Conclusions:**

There were no delayed unions or refractures among patients after carrying out a procedure in which bone grafts were routinely performed, combined with adequate periods of immobilization and non weight-bearing. These findings suggest that this procedure may be useful option for athletes to assuring return to competition level.

## Background

Proximal fifth metatarsal metaphyseal-diaphyseal fractures occur commonly in athletes, and are often characterized by difficulty of bone union [1-3]. Although treatment options include both conservative treatment and surgery, the latter is often recommended for athletes due to the long treatment period and high incidence of complications associated with conservative treatment [3,4]. However, complications such as refractures and delayed unions have been reported even when surgery is performed [5-7], and there is no consensus regarding the optimal surgical procedure.

We have treated these fractures by inserting a largest cannulated cancellous screw that would fit within the medullary canal with the goal of enabling athletes to return to play after seven to eight weeks without setting weight-bearing restrictions. However, there were more than a few athletes who experienced delayed unions or refractures. Accordingly, we modified the procedure by combining largest screw insertion and bone autografting to attain secure bone union, and reduce the risk of delayed unions and refractures. Moreover, we established post-operative periods of immobilization and non weight-bearing. The purpose of this study was to review the clinical results of our surgical treatment of proximal fifth metatarsal metaphyseal-diaphyseal fracture. The hypothesis of this study was that the intramedullary fixation combined with bone autografting could lead to high bone union rate.

## Methods

The clinical charts and radiographic studies of 15 patients who underwent surgical treatment for proximal fifth metatarsal metaphyseal-diaphyseal fractures between 2000 and 2011 were reviewed. The study protocol and publication were approved by our institutional review board and were registered with our institutional review board with the register number 112-02.

Patient demographic data are reported in Table 1. The mean follow-up period after surgery was 4.1 years (range, 1.1 to 9.8). In five patients, final follow-up was obtained by phone survey with the athlete confirming full function, the absence of pain in the operative foot, and that no additional procedures were necessary. The mean age of the 14 male and one female patient was 20.2 years (range, 16 to 31). The numbers of patients playing soccer, rugby, and handball were 13, one, and one, respectively, with all playing at the competitive level. Eight patients were professional soccer players.

**Table 1:** Patient demographic and clinical data

**Sex**	**Age, year**	**Side**	**Sports**	**Torg classification**	**Fracture site**	**Time of bone union, day**	**Time of full return, day**	**Follow up period, year**	**Complication**
M	23	Right	Soccer	nonunion	Proximal diaphyseal fracture	63	84	7.3	
M	19	Left	Soccer	delayed union	Proximal diaphyseal fracture	47	75	9.8	
M	25	Right	Soccer	delayed union	Proximal diaphyseal fracture	56	84	7.0	Screw tip stress fracture
M	18	Right	Soccer	delayed union	Jones fracture	56	64	4.1	
M	19	Left	Soccer	nonunion	Jones fracture	70	82	6.7	Screw tip stress fracture
M	18	Left	Soccer	nonunion	Jones fracture	84	120	4.3	Thermal necrosis of skin
M	21	Right	Soccer	nonunion	Proximal diaphyseal fracture	84	98	3.9	
M	17	Left	Soccer	delayed union	Proximal diaphyseal fracture	48	96	3.2	
M	23	Right	Soccer	acute fracture	Proximal diaphyseal fracture	56	71	3.2	
F	17	Right	Handball	acute fracture	Proximal diaphyseal fracture	42	82	3.0	
M	17	Right	Soccer	delayed union	Proximal diaphyseal fracture	42	84	2.9	
M	16	Left	Soccer	delayed union	Jones fracture	62	76	2.0	
M	31	Left	Rugby	nonunion	Jones fracture	56	90	2.0	
M	21	Right	Soccer	delayed union	Jones fracture	54	82	1.3	
M	18	Left	Soccer	nonunion	Proximal diaphyseal fracture	60	82	1.1	

The fracture type according to classification of Torg et al. [8], fracture location [9], the diameter of the inserted screw, time to bone union following surgery, time to initiation of running, time to return-to-play, rate of return-to-their previous level of athletic competition, and complications were investigated.

The fracture type was divided into three types based on radiographic findings and clinical progression: acute fractures that show a clear and sharp fracture line without a prior history of pain or injury; delayed unions with a prior history of injury or fracture that exhibit osteosclerosis accompanied by new bone growth or bone resorption at fracture lines; and nonunions that show medullary cavity occlusion along with repeated injury, recurrent pain, and marked osteosclerosis around the fracture site [8].

The fracture site was classified into two types: Jones fracture and proximal diaphyseal fifth metatarsal fracture [2,9,10]. A Jones fracture was defined as a fracture between the proximal diaphysis and the metaphysis of the fifth metatarsal, where the fracture line did not reach the distal areas of the fourth and fifth metatarsal joints. A proximal diaphyseal fifth metatarsal fracture was defined as a fracture in which the fracture line was located in the proximal diaphysis more distal than the fracture line in a Jones fracture. An avulsion fracture of the tuberosity was not included in this study.

Indication for surgery was based on development of a complete fracture despite the fracture type according to classification of Torg et al. For incomplete fractures, the patients were allowed to continue competing without restriction if they were able to do so.

**Figure 1:**
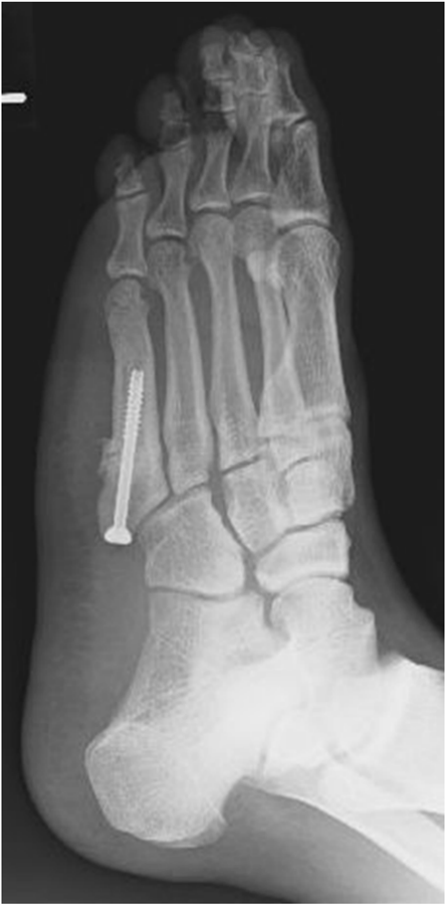
**X-ray immediately following surgery.** The fracture site was curetted, a cannulated cancellous screw with a diameter of 5 mm was inserted, and autologous bone was grafted.

The procedure was performed in the lateral or supine position under fluoroscopy. For the supine position, a pillow was placed under the buttocks and the affected limb was rotated internally. Pneumatic tourniquet was used in all surgeries. First, a skin incision was made laterally to the fracture site, and the fracture site was freshened with a curette. A small incision was placed proximal to the fifth metatarsal. Careful blunt dissection was carried out to the area proximal to the fifth metatarsal. Care was taken to avoid or protect the sural nerve. An assistant inverted the ankle joint, and a surgeon inserted a guide pin for the titanium cancellous screw (ACE cannulated cancellous screw, Japan Medical Dynamic Marketing, Tokyo, Japan). Drilling was performed after confirming the satisfactory position of the guide pin under fluoroscopy. The target screw diameter was more than 5 mm, and the screw with the largest possible diameter was inserted. In the case that the medullary canal was narrow, drilling began with a thin bit appropriate for a 4-mm screw, and the diameter was gradually increased to that of the screw scheduled for insertion. If the fracture site showed osteosclerosis, we removed as much of this as possible by drilling. The screw length was determined such that the threads of the partially threaded screw were past the fracture site and the screw heads were countersunk. At the time of screw insertion, the screw head was completely buried. Finally, the autologous bone harvested from the proximal tibia medially to the tibial tuberosity was grafted to the fracture site opened in advance (Figure 1).

**Figure 2:**
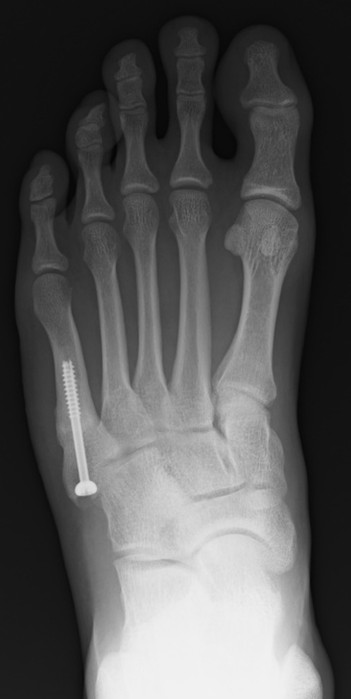
**X-ray images from four directions to confirm bone union.** Anteroposterior radiograph.

**Figure 3:**
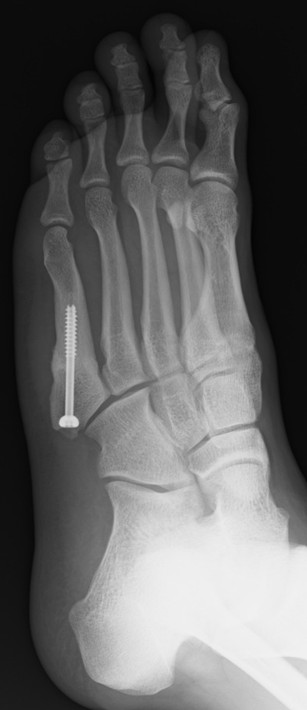
**X-ray images from four directions to confirm bone union.** Oblique radiograph at 30° internal rotation.

**Figure 4:**
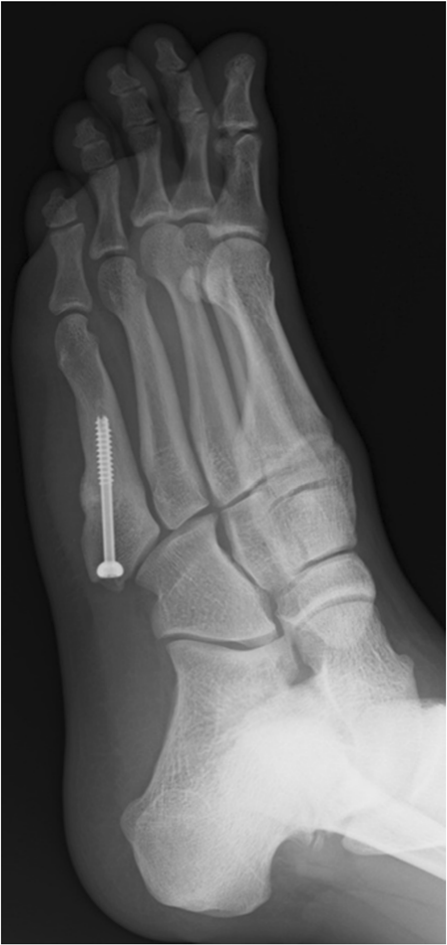
**X-ray images from four directions to confirm bone union.** Oblique radiograph at 45° internal rotation.

**Figure 5:**
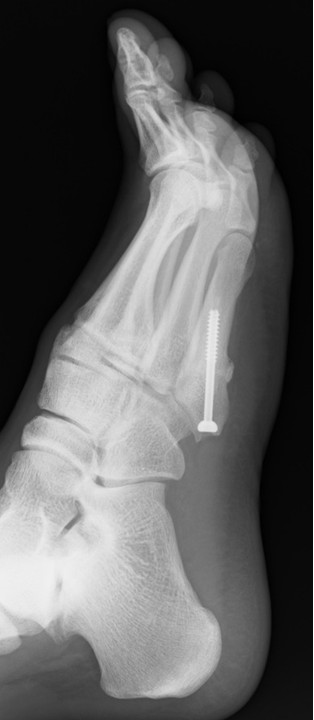
**X-ray images from four directions to confirm bone union.** Oblique radiograph at the maximum external rotation.

Postoperatively, patients remain non weight-bearing in a splint or cast for two weeks and non weight-bearing without external stabilization for an additional two weeks. Full weight-bearing was allowed six weeks postoperatively. Radiographic bone union was evaluated from four directions at follow up: in the anteroposterior, 30° internal rotation, 45° internal rotation, and the maximum external rotation (Figures 2, 3, 4 and 5). Bone union was considered to have occurred when cortical bone continuity was obtained in all directions. At this point, runnning with custom-made shoe insoles was allowed. Subsequently, the intensity was gradually increased and return to full activity was permitted.

## Results

Two patients had acute fractures, seven had delayed unions, and six had nonunions according to classification of Torg et al.

Based on fracture location, six patients had Jones fractures, and nine had proximal diaphyseal fifth metatarsal fractures.

For 14 procedures, 5-mm-diameter screws were inserted. For one procedure, 4-mm-diameter screw was inserted because of the small outer diameter of fifth metatarsal.

The mean time to the radiographic evidence of complete bone union was 8.4 weeks (range, 6 to 12), and the mean time to initiation of running was 8.8 weeks (range, 7 to 12). The mean time to return-to-play was 12.1 weeks (range, 9 to 17). All patients returned to their previous level of athletic competition.

**Figure 6:**
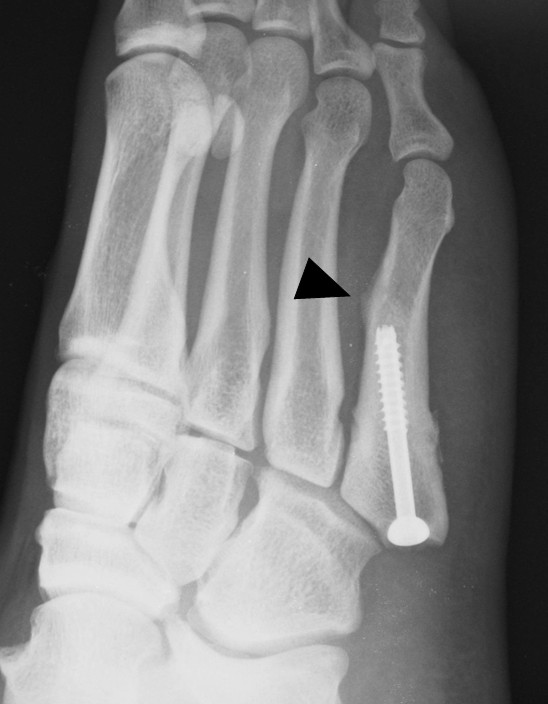
**Stress fracture at screw tip (black arrow head).** A thickened bone cortex can be seen at the dorsal bone cortex of the screw tip.

There were no refractures during the follow-up period. Diaphyseal stress fractures at the distal tip of the screw occurred in two patients. In both patients, the original fracture had unioned and the screw tip was in contact with the dorsal bone cortex of the diaphysis (Figure 6). For one patient, stress fractures at the screw tip were diagnosed at 19 weeks post primary surgery, and it was unioned by replacing the screw with a shorter one. The patient returned to competitive sports at eight weeks after revision surgery. The other patient was diagnosed at 14 weeks postsurgery. The patient could continue to play without pain wearing a shoe insole, and the stress fracture unioned without time away for the diaphyseal stress fracture at the screw tip. One patient experienced a thermal necrosis of skin, presumably caused by the reaming heat from the pre-drilling for screw insertion. The wound healed after three debridement procedures. At 17 weeks post surgery, the patient returned to competitive level before complete closure of skin necrosis. None of the patients complained of the site from which bone was harvested.

## Discussion

To the best of our knowledge, our report is the first in which intramedullary screw fixation with routine bone autografting was performed during the primary surgery. Although the current gold standard for treatment is intramedullary screw fixation, delayed unions and refractures with this procedure are not uncommon [5,7,8,11,12]. Bone autografiting could solve the problem because it is osteoconductive, osteoinductive, and provides osteogenic cells [13,14].

The intramedullary screw fixation without bone grafting had the advantage of early return-to-play. In the previous literature, mean time to return-to play was reported 7.5 to 8.5 weeks [4,15,16]. Although positive outcomes have been reported, considerable frequency of complications have been reported [5,13]. Larson et al. reported 40 percent of failure rate in the procedure, and especially a higher proportion of elite athletes had high incidence of failure [5].

The poor blood supply to the metaphyseal region of the proximal fifth metatarsal is considered the factor to lead to a deficiency in fracture healing factors [17,18]. This insufficiency in biology may be indirectly addressed by the application of bone autografting [19]. Hunt and Anderson used bone grafts in combination with insertion of a screw with the greatest possible diameter in 15 revision surgeries and six primary surgeries of nonunion or refracture [19]. They reported that all patients returned to competition, but the mean time of return-to-play was 12.3 weeks. The period of non weight-bearing and immobilization of the affected area tends to be longer than in other reports due to the bone autografting. However, we believe that bone autografting is an effective option to obtain the secure bone union in the surgical treatment of proximal fifth metatarsal metaphyseal-diaphyseal fracture.

Although several authors considered that routine bone autografting to the fracture site was not necessary, especially in acute fracture according to classification of Torg et al. [15,20], we performed routine bone autografting for all fracture types. Glasgow et al. reported 11 patients with failure of surgically managed fractures, and six of 11 patients were acute fracture [13]. Wright et al. analyzed six patients with treatment failure. In the study, all patients had acute fracture at the time of primary surgery [7]. We considered that delayed unions and nonunions often occur in acute fracture according to classification of Torg et al. as well as delayed union or nonunion according to Torg’s classification. Thus, we consider that routine bone grafting for all fracture types is effective.

In the present study, while delayed unions or nonunions were not observed, some patients experienced stress fractures at the screw tips and thermal necrosis of skin due to reaming heat. In both patients developed a stress fracture at the screw tips, the screw tip was in contact with the dorsal bone cortex. In one patient, the dorsal convex curvature was large, and the screw tips inevitably contacted the dorsal bone cortex in order to ensure that the screw threads completely crossed the fracture site. In such situations, it is important to insert the screw so that aligns as closely as possible with the bone axis to avoid excessive stress on the dorsal bone cortex side. One solution is to place the screw insertion point at a sufficiently dorsal position, and to insert the screw toward the sole side. However, given that the screw head is positioned dorsally in this procedure, for a soccer player, pain may appear in the area when kicking. Hence, it is necessary to countersink the screw head sufficiently. Preoperative evaluation of the morphology of metatarsals is crucial. When stress fracture at screw tip occurs, replacing the screw contacting with cortex is a foundational rule of treatment. In the present study, however, the screw tip stress fracture unioned without screw replacing in one patient. If the patient could continue playing with shoe insole, we might treat stress fracture conservatively. But, in the case which patients feel pain, there must be no wavering of replacing screw because the stress fracture has the potential to develop complete fracture.

Pre-drilling is required when inserting the cancellous screw and this can lead to thermal necrosis. Heat generated when drilling hard sclerotic bone or from friction between the bent guide pin and the drill is a likely cause. We have been cooling the skin with cold water during drilling ever since this patient, and have not observed skin burns thereafter. Recently, we consider releasing the pneumatic tourniquet effective for preventing thermal necrosis, and routinely release the pneumatic tourniquet before reaming. Blood flow is poor for the skin around the metatarsals. Once a thermal necrosis occurs, it may require a prolonged healing period.

There were several limitations in this study. There is no comparison group and the study sample was relatively small. Another limitation is that the follow-up period is less than two years in two patients. Although most refractures were diagnosed within one year after surgery in previous studies [11,21], refractures could occur at any time. The short follow-up period is an important limitation of this study. Finally, two different fracture locations were analyzed in combination. Although several authors reported the proximal diaphyseal fifth metatarasal fracture is more difficult to obtain satisfactory outcomes than Jones fracture [1,22], the largest study in comparison between two fracture locations revealed no difference in clinical and radiographic outcomes [9]. We also consider that the treatment of Jones fracture is challenging as well as proximal diaphyseal fifth metatarsal fracture. Acknowledging these weaknesses, we believe that this series contributes valuable information for surgical treatment of challenging proximal fifth metatarasal metaphyseal-diaphyseal fracture because of the encouraging outcome.

We have reported the outcomes of intramedullary screw fixation combined with bone autografting. Consistent with our aims, the results suggest that the procedure is useful for preventing delayed unions and refractures. However, caution is required as we have experienced other complications.

## Conclusions

No delayed union or refractures occurred in our procedure that combines bone autografting with intramedullary insertion of a screw with the largest possible diameter, and by confirming bone union with radiography before approving an athlete’s return-to-play. These results suggest that this procedure may be effective option for assuring return-to-play.

## Competing interests

The authors declare that they have no competing interests.

## Authors’ contributions

ST conducted the retrospective study and drafted the manuscript. SN developed the surgical procedure. HI, YS, MS and AH helped complete the manuscript. All authors read and approved the final manuscript.
